# A Rare Culprit of Simultaneous Arteriovenous Thromboembolism: Acute Viral Perimyocarditis

**DOI:** 10.1155/2019/5361529

**Published:** 2019-01-08

**Authors:** Christoph Sossou, Tobi Ogundare, Ogechukwu Chika-Nwosuh, Amee Sodha, Christopher Nnaoma, Cynthia McKinney, Jose Bustillo

**Affiliations:** ^1^Internal Medicine Resident, Newark Beth Israel Medical Center, Newark, NJ, USA; ^2^Teaching Attending Physician, Newark Beth Israel Medical Center, Newark, NJ, USA; ^3^Medical Student, St. George's University School of Medicine, Grenada; ^4^Associate Program Director, Internal Medicine, Newark Beth Israel Medical Center, Newark, NJ, USA

## Abstract

Simultaneous arteriovenous embolism is extremely rare. Herein, we present a rare case of systemic arteriovenous emboli in a healthy 33-year-old male after an episode of acute viral perimyocarditis. The culprits are postulated to be viral-induced myocardial necrosis and resulting proinflammatory state in the setting of negative malignant, autoimmune, genetics, and chronic infectious conditions. The patient was successfully managed with guideline-directed medical therapy and safely discharged to a subacute rehabilitation facility.

## 1. Case Introduction

Simultaneous arteriovenous thromboembolism is an extremely rare phenomenon. This is a case of a 33-year-old male with dual cardiac chambers thrombi, complicated by arteriovenous thromboembolism, four months after an acute episode of viral perimyocarditis.

## 2. Case Description

A 33-year-old African-American man (BMI 22) presents to the emergency department of our facility with progressive dyspnea with mild exertion and bilateral lower extremity (LE) edema associated with paresthesia and pain. Four months prior, he was treated for viral perimyocarditis complicated by new onset heart failure with reduced left ventricular ejection fraction (LVEF ~ 30–35%).

**Figure 1:**
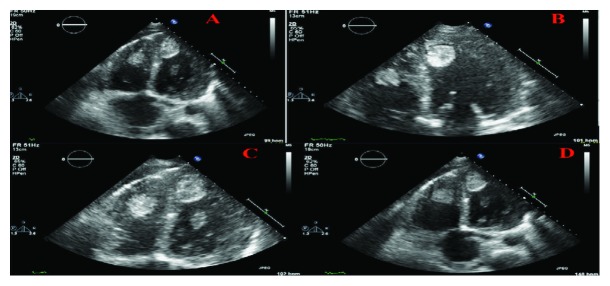
Transthoracic echocardiography, apical 4-chamber views, and multiple pedunculated 2-3 cm echodensities in the right and left ventricles consistent with intracardiac thrombi (a–d): moderate left ventricular hypertrophy (a), borderline LV dilatation (b), right ventricle appears moderately dilated (c), and intra-atrial septal deviated to the left (d). There is a small pericardial effusion.

**Figure 2:**
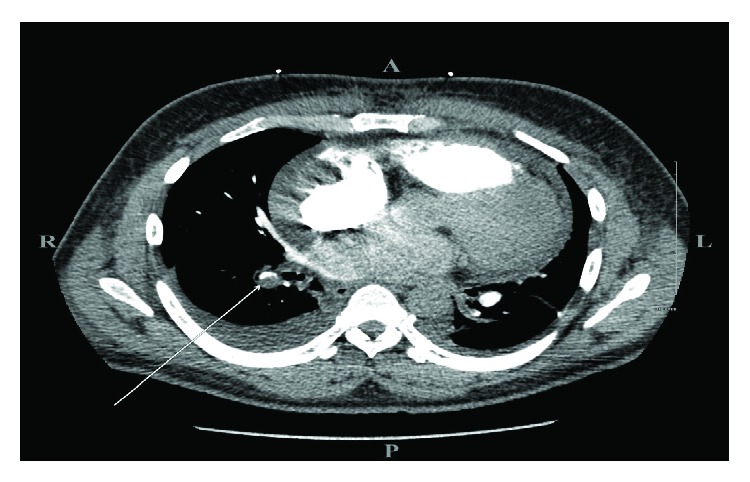
A contrast-enhanced chest computed tomography with pulmonary embolism protocol: large filling defect within the distal right lobar pulmonary artery, with thrombus extending into the right lower lobe branches (white arrow). The heart is markedly enlarged with a moderate pericardial effusion. Bibasilar consolidations and areas of linear atelectasis versus scarring/fibrosis at both lower lobes. Bilateral pleural effusions, larger on the right than the left. There is no pneumothorax or pneumomediastinum.

**Figure 3:**
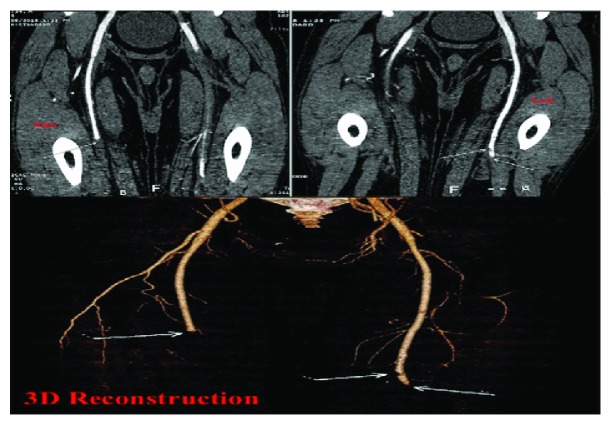
A computed tomography angiogram of bilateral lower extremity: severe occlusive bilateral superficial femoral arteries at the level of the mid thighs and occlusion of the right internal iliac artery. Mild reconstitution is seen on the right side anterior tibial artery with no flow in it seen distal to the ankle. No reconstitution is seen on the left side. No radiographic evidence of collateralization.

**Figure 4:**
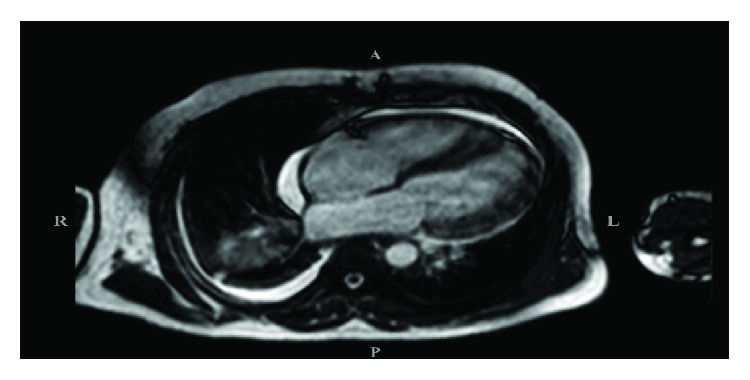
Contrast-enhanced cardiac MRI: contrast late enhancement study shows diffuse patchy contrast late enhancement mostly involving mid- and epicardial layer of myocardium, more at the midlateral wall and inferior wall which can be consistent with myocarditis. Small pericardial effusion.

He was noted to be afebrile, tachycardiac to (110 s) BPM, tachypneic (20 s) Br/min, pulse oximetry of 93% on room air, normotensive (110/70 mmHg) and oxygen saturation is 93% breathing ambient air. Cardiopulmonary exam revealed jugular venous distention, diffuse bilaterally crackles, bilateral pitting LE edema up to the knee, reduced femoral, and dorsalis pedis pulses. The rest of the exam was otherwise unremarkable. Initial laboratory findings were significant for neutrophil-predominant leukocytosis (WBC 19 × 10^3^ mcL), elevated brain natriuretic peptide (BNP 2506 pg/mL), troponin-I 0.48 ng/mL (normal < 0.03), D-dimer 6.6 *μ*g/mL (normal high < 0.46), erythrocyte sedimentation rate 110 mm/Hr (normal high 15), and C-reactive protein 12.7 mg/dL (normal high 0.99). Transthoracic echocardiogram showed severe global hypokinesis, moderate-to-severe systolic dysfunction, paradoxical septal motion, and multiple biventricular pedunculated mobile echodensities (about 2-3 centimeters in size) consistent with thrombi with trace pericardial effusion (Figure 1). Contrast-enhanced pulmonary embolism (CTPE) study revealed right lobar pulmonary embolus and bilateral small pleural effusions (Figure 2). Lower extremity computed tomography angiogram (CTA) revealed bilateral occlusion of the superficial femoral, popliteal, posterior tibial, peroneal, and anterior tibial arteries (Figure 3). Patient underwent urgent embolectomy and thrombectomies, with successful restoration of adequate inflow. Contrast-enhanced cardiac MRI revealed diffuse patchy late enhancement of the epicardial layer of the myocardium and small pericardial effusion consistent with perimyocarditis (Figure 4). He was managed with intravenous furosemide, heparin drip, successfully transitioned to rivaroxaban, and safely discharged to a subacute rehabilitation facility, with significant improvement noted on subsequent follow-ups.

## 3. Case Discussion

Intracardial thrombi are common in patients with severe left ventricular systolic dysfunction, but multiple intracardial thrombi leading to simultaneous arteriovenous thromboembolic are extremely rare moderately reduced left ventricular ejection [1, 2]. The underlying mechanisms for intracardiac thrombogenesis have been postulated as a complication of blood stasis, hematological hypercoagulability, inflammatory conditions (i.e., viral myocarditis, vasculitis), cardiac dysrhythmias, myocardial infarction, cardiomyopathic states, or genetic conditions and deficiencies [1–6]. This is a unique case because unlike previously reported cases of multicardiac chamber thrombi, our patient only had a moderately reduced LVEF (30–35%) but with a higher clot burden. Concerning the possibility of hypercoagulability, a thrombophilia screen was performed before the initiation of heparin, revealing normal antithrombin III level, protein S, and protein C along with negative anticardiolipin, lupus anticoagulant, and beta 2 glycoprotein antibody. Genetic tests for factor V Leiden and prothrombin gene mutation were also unremarkable. Possible explanation in our case includes viral-induced myocardial necrosis (elevated troponin I) prompting chronic inflammatory state (elevated ESR and CRP). Acute viral perimyocarditis causes myocardial necrosis, which leads to chronic inflammatory state and stimulates systemic cytokines circulation, causing further endothelial injury, platelet activation, thrombi generation, and propagation. Acute viral myocarditis is also associated with increased tissue factor (TF); a low molecular weight glycoprotein initiates the clotting cascade and is considered to be a major regulator of coagulation, hemostasis, and thrombosis [1, 7].

The optimal management of intracardial thrombi remains uncleared [8]. Warfarin, the antithrombotic therapy of choice for intracardial thrombi management, has unpredictable pharmacodynamics and pharmacokinetics, making it an unattractive option. Thus, there is limited data on direct oral anticoagulant (DOAC) usage in intracardial thrombi; DOAC has a better safety profile compared to warfarin [4, 8, 9]. Direct oral anticoagulation (i.e., rivaroxaban) has been extensively studied in deep vein thrombosis, nonvalvular atrial fibrillation, and pulmonary embolism and has been used for many years for the prevention of stroke in patients with these conditions [8, 9]. Hence, it is reasonable to assume that if patients are compliants, DOAC could be a safer and effective alternative to warfarin in intracardial thrombi management. Our patient was optimized with guideline-directed heart failure therapy and safely discharged to subacute rehabilitation center on rivaroxaban 15 mg twice daily for 21 days and 10 mg once daily thereafter. The patient has clinically improved and is free of any complication as noted in subsequent follow-up visits. This case illustrates a rare complication of acute viral perimyocarditis and highlights the need for a randomized controlled clinical trial involving the use of DOAC in patients with intracardiac thrombi.

## 4. Conclusion

This case illustrates a rare complication of acute viral perimyocarditis: intracardial thrombi complicated by simultaneous arteriovenous thromboembolic events. Besides warfarin, there are few data to guide clinicians' decision regarding choice of antithrombotic therapy in intracardial thrombi management. Our case demonstrates that DOAC is a safe and effective antithrombotic option. Nonetheless, randomized clinical trials are needed before DOAC can be universally recommended as the mainstay of antithrombotic therapy in intracardial thrombi management.
